# Nail Regeneration of an Allen III Fingertip Amputation After a Dog Bite Using the Semi-occlusive Dressing Technique: A Case Report and Literature Review

**DOI:** 10.7759/cureus.17068

**Published:** 2021-08-10

**Authors:** Dimitris J Georgoulis, Dimitra Melissaridou, Ioannis Zafeiris, Panayiotis J Papagelopoulos, Olga D Savvidou

**Affiliations:** 1 First Department of Orthopedics, Attikon University Hospital, Athens, GRC; 2 First Department of Orthopaedics, Attikon University Hospital, Athens, GRC

**Keywords:** fingertip amputation, semi-occlusive dressing, conservative, animal wound bites, allen iii injury

## Abstract

There is ongoing controversy regarding the best treatment of fingertip amputations, conservative treatment with secondary healing or surgical intervention. Healing by secondary intention has been proven to offer satisfactory recovery and function. More extensive wounds are treated surgically. However, even in Allen III and IV amputations, there is a lack of evidence to support enhanced healing and function of fingertips after surgical treatment compared to conservative management. Regarding fingertip amputations after animal bites, thorough debridement is the preferred treatment due to various micro-organisms, while there is no consensus about the primary closure of the wound.

Inclusion cri­teria are complete amputations even with bone involvement at all levels. Exclusion criteria are skeletonized distal phalangeal bone, not surrounded by soft tissues, joint involvement and exposed tendon. It offers complete regeneration of the fingertip without signs of infection, even in animal bites wounds. There are few reports in the literature regarding the semi-occlusive dressing for treating fingertip amputations-only one report uses this technique after an animal bite in a two-year-old girl.

In this case report, an Allen III fingertip amputation caused by a dog bite in a 64-year-old female was managed successfully using the semi-occlusive dressing technique. At the final follow up three months after the injury, the aesthetic results were satisfactory. The fingertip with the nail complex was almost normal with no nail hook deformity. The pad skin regenerated with no signs of infection. The functional results were excellent, with no joint stiffness or disability. The sensibility was satisfactory with two-point discrimination of 4 mm, and there was no tenderness, cold intolerance, or neuroma. The patient was satisfied and able to participate in all daily activities.

The semi-occlusive dressing technique is an alternative treatment option for Allen III fingertip amputations after animal bites. It promotes regenerative healing, and despite bacterial colonization, no infection has been reported.

## Introduction

According to the degree of injury based on Allen classification, Fingertip amputations are classified from Type I to Type IV [1]. Although there is no consensus regarding the best treatment option [2], wounds up to 1-2 cm^2^ are managed conservatively. In contrast, larger are managed with different surgical techniques, including revision amputation, local and pedicle flaps, cross finger flaps, composite graft, autograft coverage, reimplantation, and microsurgical replantation: the surgeon's educational background, prior experience, facility and cultural environment. Revision amputation with shortening the exposed bone to make the primary wound closure possible is the most common treatment since it is effective with good outcomes. However, although wound repair will provide quick wound healing, it may cause significant deformity and functional loss of the hand function or finger [3].

Non-surgical treatment using the semi-occlusive dressing has received little attention in the literature, despite considerable evidence supporting that semi-occlusive dressing yields similar, if not superior, results [4]. Illingworth et al. in 1974 reported a simple, relatively painless and effective method of treating trapped fingers in children [5]. They discovered the fingertip's capacity for self-regeneration with the occlusive wound dressing, which creates a contained environment around the amputation. Mennen et al. in 1993 reported similar encouraging results using a film coverage [6]. Further studies showed that the tips of human fingers regenerate de novo and semi-occlusive dressing technique, with different kinds of film dressing occlusions and silicone finger cap, had superior results compared to surgical intervention [7,8]. This regenerative phenomenon is more frequently seen in young children [5], although there is extensive data for fingertip regeneration after amputations even in adults [7,8]

Animal bites are a typical injury pattern. Dog bites are also prevalent. Normally, a dog bite is initially recommended for wound lavage and keep the wound open [9]. Also, there are high chances of progression to cellulitis and osteomyelitis with any conservative management of dog bites.

To the best of our knowledge, there are few reports in the literature regarding the regeneration of a fingertip amputation after an animal bite. Using the semi-occlusive dressing technique, this case report achieved the regeneration of a fingertip Allen III amputation caused by a dog bite without signs of infection.

## Case presentation

**Figure 1:**
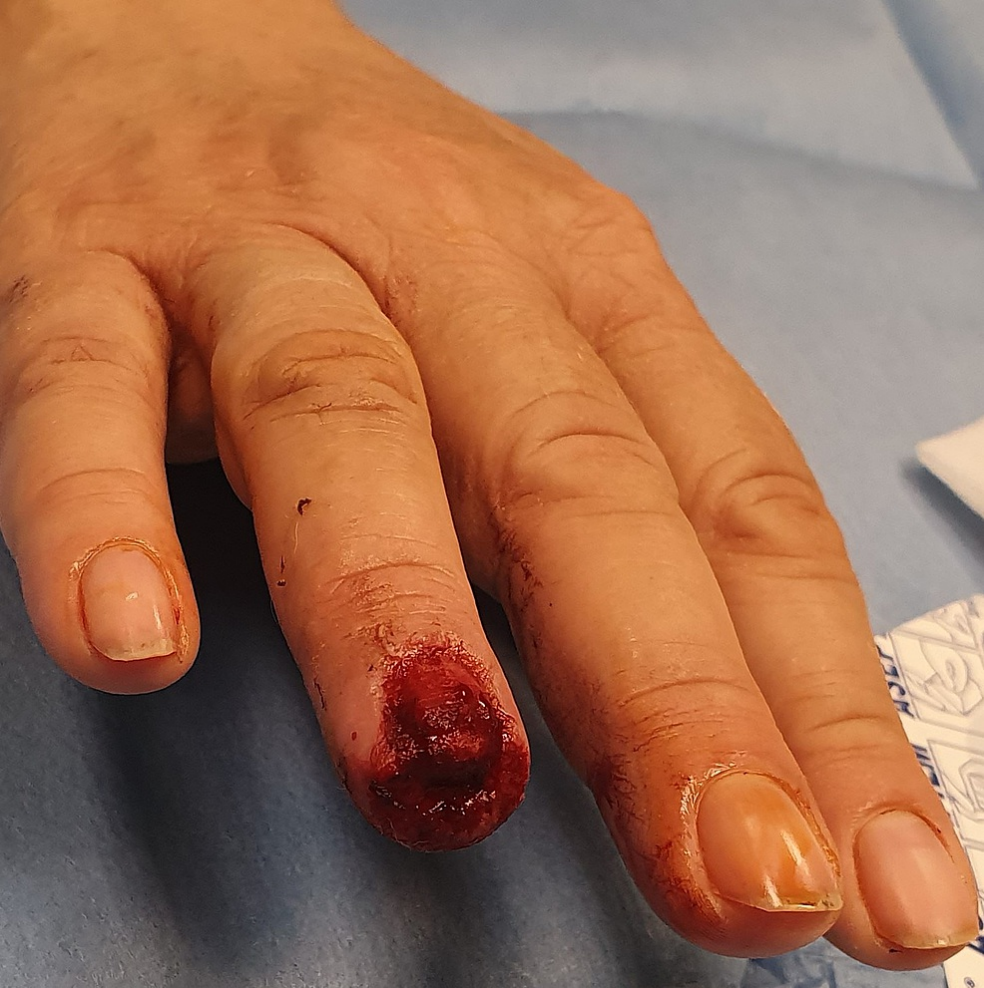
Fingertip amputation, Allen III of the ring finger of the right hand of a 64 years old female.

**Figure 2:**
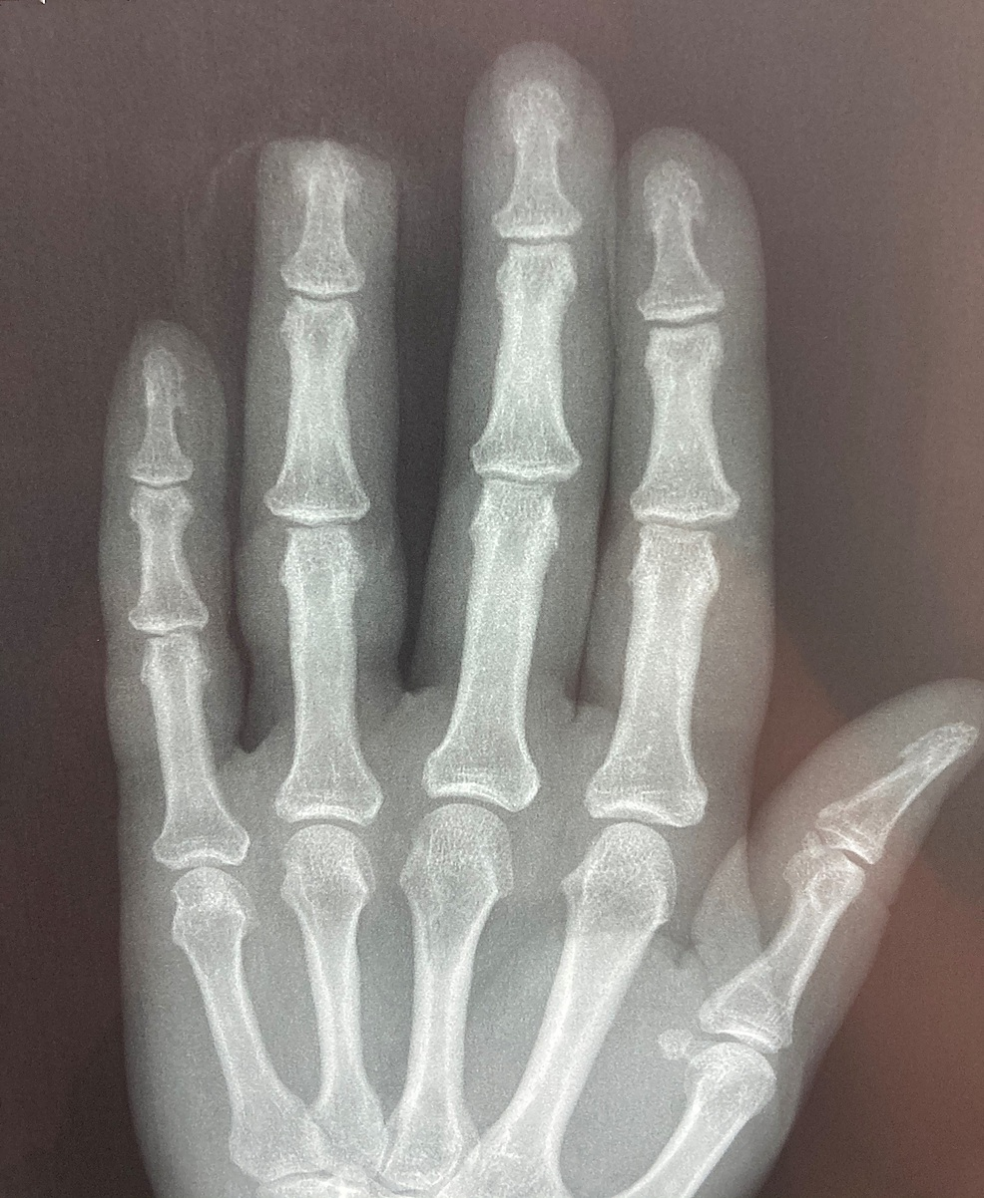
Anteroposterior radiographs of the hand showing the soft tissue injury of the ring finger.

A 64-year-old female, with an Allen type III amputation of her right ring finger, after a dog bite was examined in the Emergency department of our Hospital. Radiographs of the right hand were undertaken in order to assess bone injuries and loss (figure 1, 2). Patient informed consent taken for the data concerning her injury and treatment would be submitted for publication.

The wound was meticulous debrided and cleaned with an antiseptic solution of povidone-iodine and hydrogen peroxide 1:1 mixture and 2 litres of normal saline. A prophylactic dose of human tetanus Immunoglobulin (HTIG) 250 IU/ml was administered. An empirical antibiotic regimen with cefuroxime and clindamycin intravenously was initiated. Due to the nature of the injury and the high possibility of the wound’s contamination by micro-organisms native to the dog bite, conservative treatment with the semi-occlusive dressing technique as described by Mennen et al. [6] and Hoigne et al. [10] was decided to be used. The dog was up-to-date vaccinated and this was confirmed by laboratory evidence, so there was no need for a prophylactic regime [11]. 

**Figure 3:**
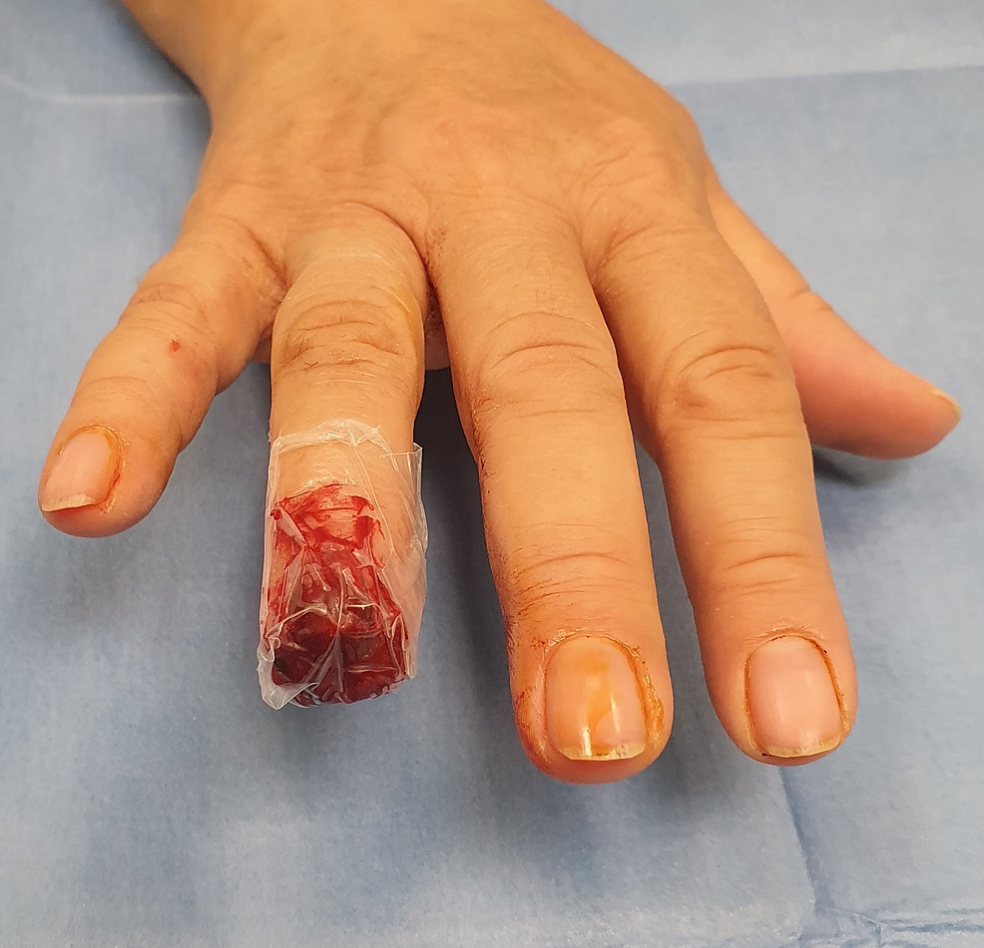
The Asepta waterproof dressing.

**Figure 4:**
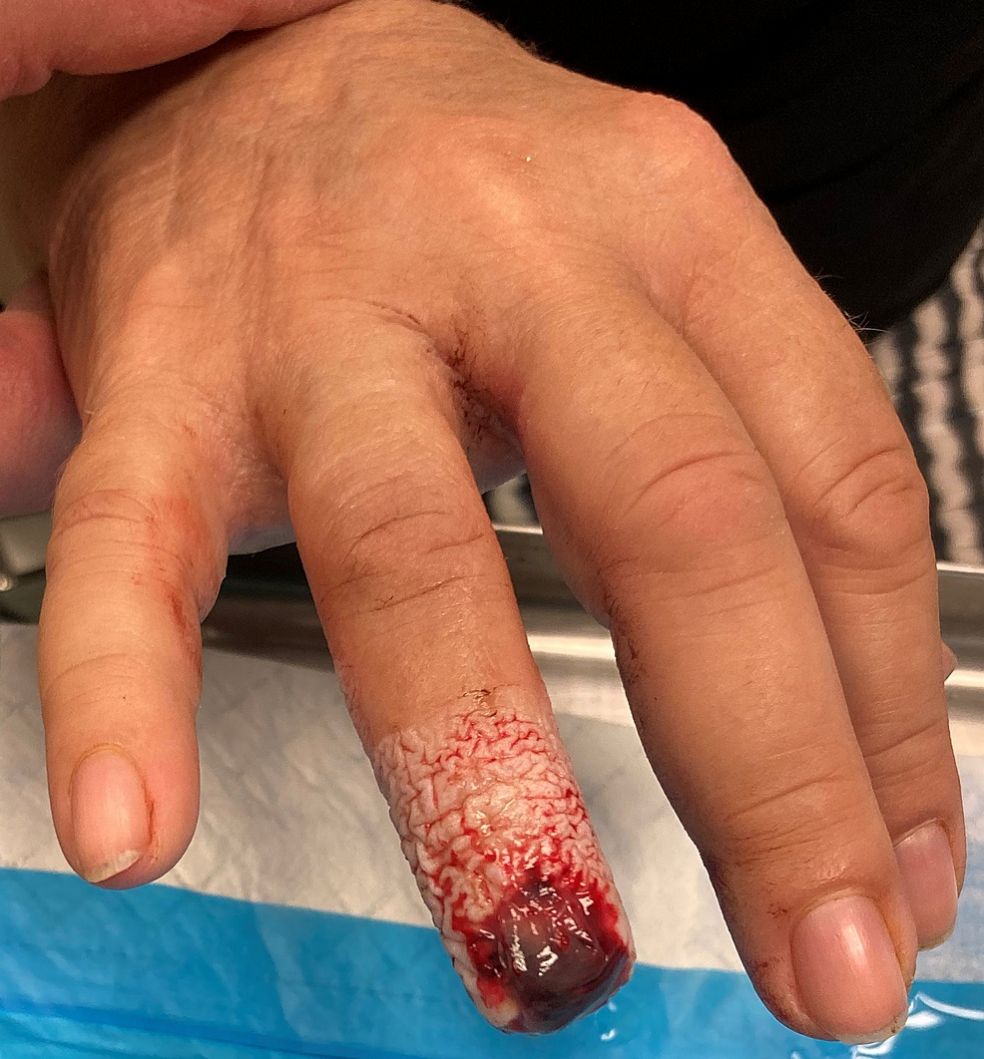
Fingertip at 4th-day post-injury.

**Figure 5:**
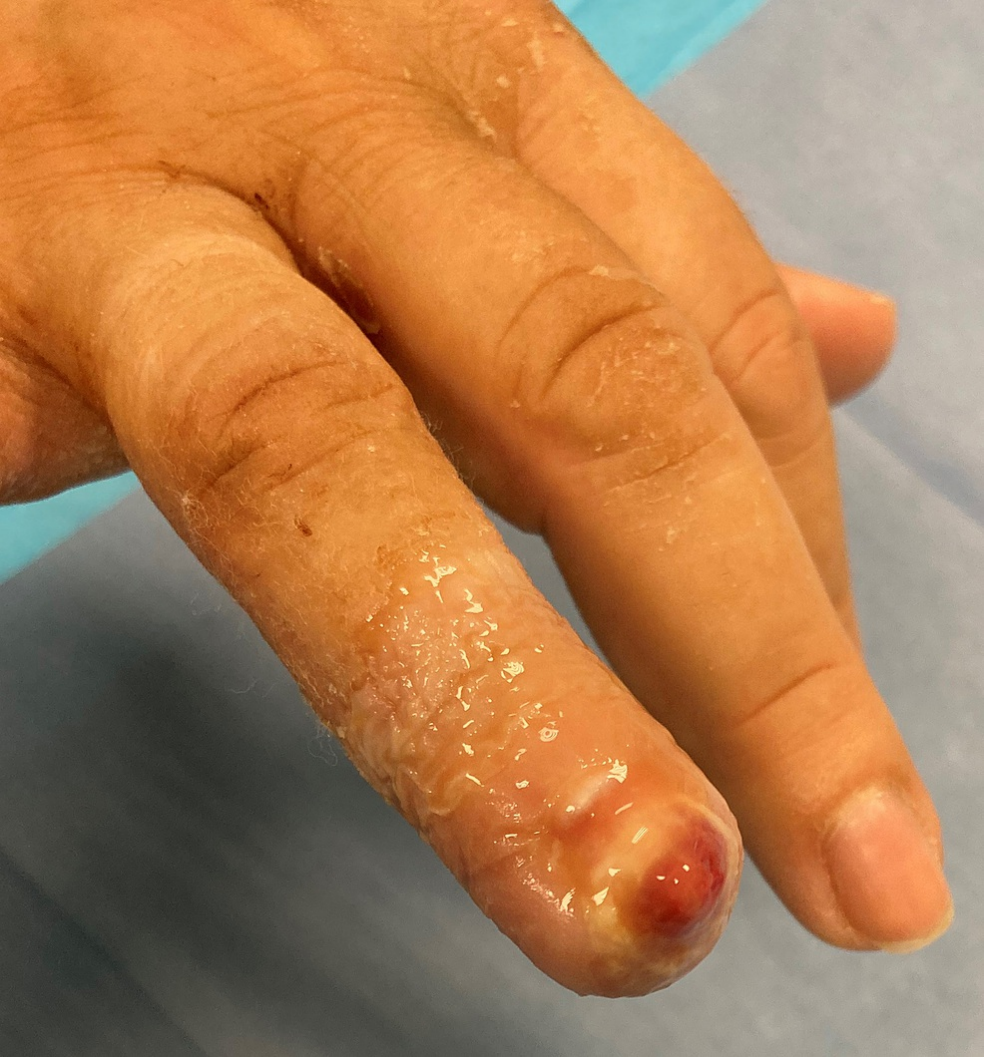
Two weeks post-injury, a new nail plate began to grow, and the fingertip was covered with granulation tissue.

**Figure 6:**
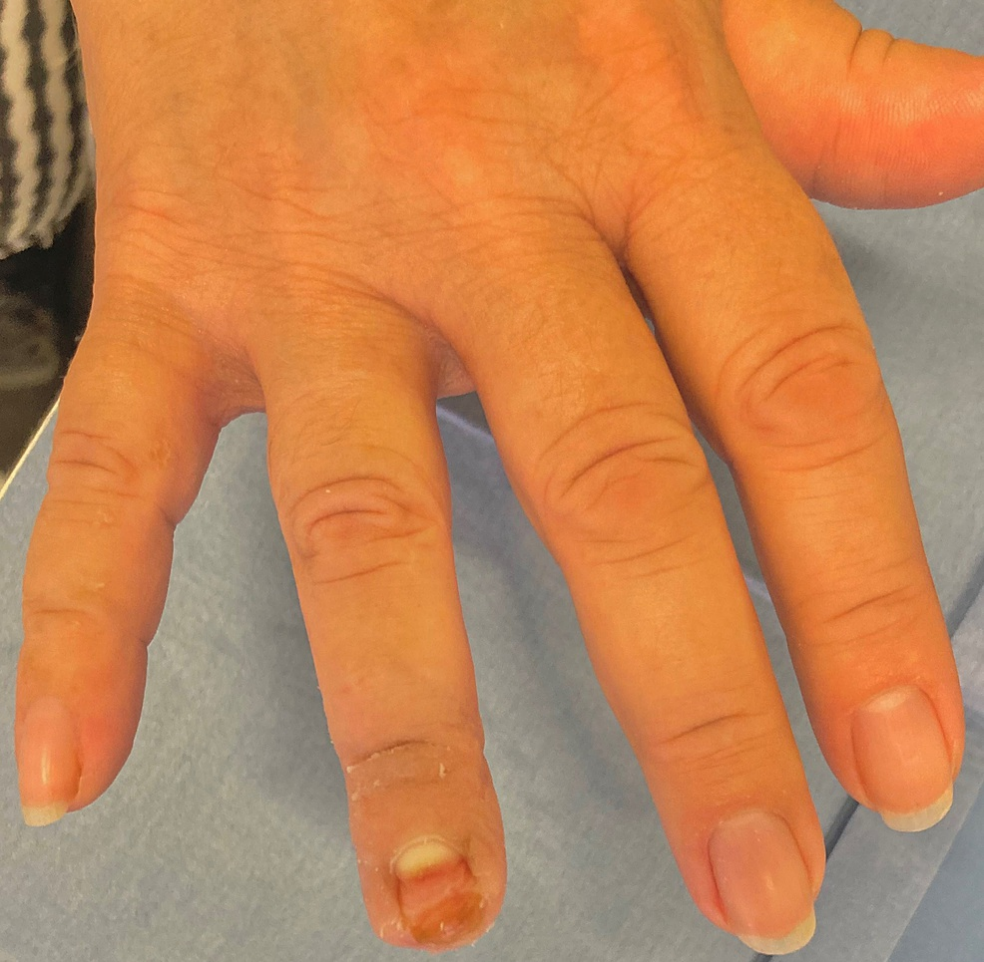
Fingertip at 4th-week post-injury, showing nail starts to grow and fingertip shape starts to resemble that of the uninjured fingers.

A transparent, waterproof adhesive dressing Asepta was used for the dressing, which is covered with a crepe bandage for protection (figure 3). An empirical antibiotic regimen was prescribed per os, cefuroxime 500 mg twice and clindamycin 300 mg thrice a day for a week. The patient was followed up in the outpatient clinic for dressing changes and wound inspection once a week. The first dressing change was performed on the 4th day (figure 4) and after that, every seventh day until day 45, when the wound ceased being productive. During the follow-up, period cleaning was omitted. The usually foul-smelling liquid film and any clots were left on the wound. No substantial pus and evidence of infection were noted. Two weeks later, a new nail plate began to grow, and the fingertip was covered with granulation tissue (figure 5). After four weeks, the granu­lation tissue grew, and the fingertip regenerated into the origi­nal fingertip shape (figure 6). After eight weeks, epithelialization was almost complete, but a slight granulation remained at the tip.

**Figure 7:**
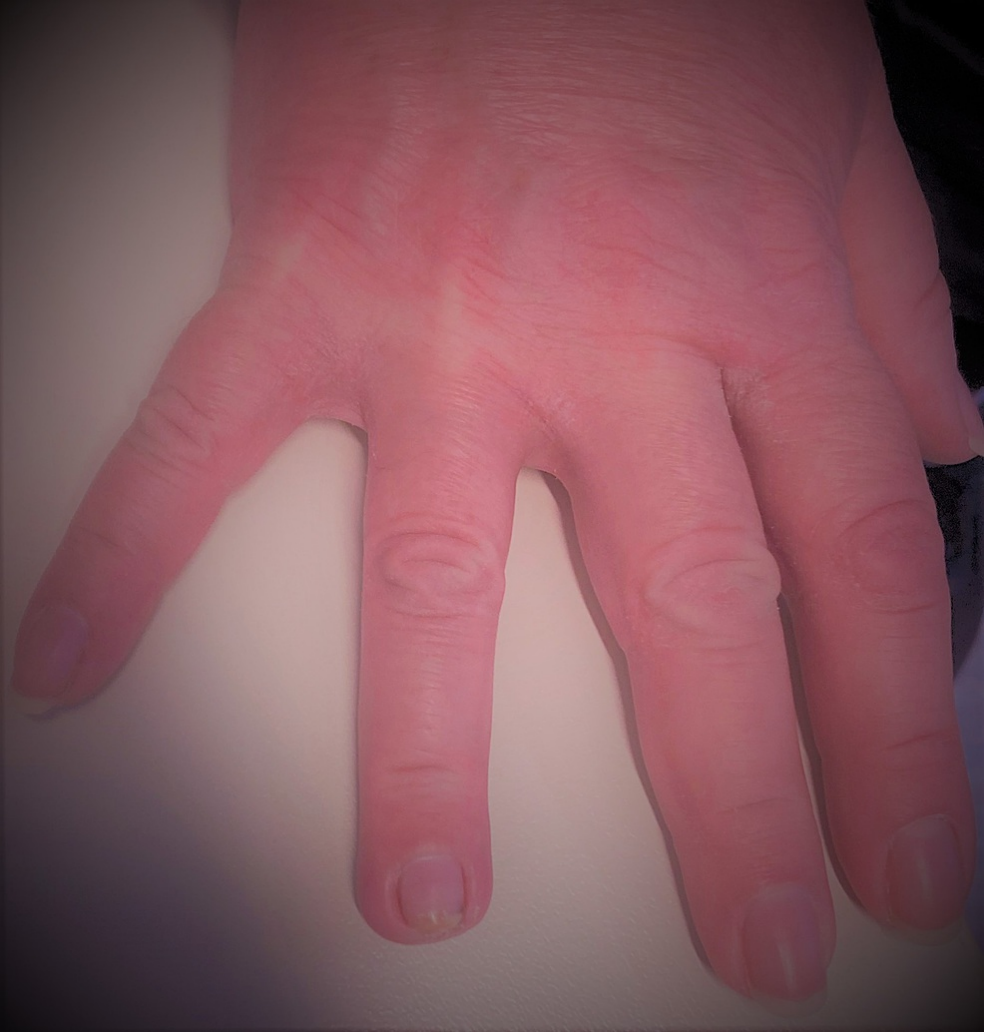
At 3 months post-injury the skin of the tip and the pulp were regenerated with scarless epithelialization and satisfactory cosmetics.

 At three months of the injury the nail complex with the nail bed, the paronychium, the eponychium, the nail fold, and the hyponychium were remodelled, without deformity and without any signs of infection. The skin of the tip and the pulp were regenerated with complete scarless epithelialization, satisfactory function and pleasant cosmetics (figure 7). The fingertip’s sensibility was satisfactory with the two-point discrimination of 4mm, while the two-point discrimination of the contralateral healthy finger was 3.4mm. There was no tenderness, cold intolerance, or neuroma, while the range of motion of the distal interphalangeal joint and the apposition of the tip towards the palm region was normal.

## Discussion

With the most common dog bites, animal bites account for 1% of emergency department visits and are critical issues in health care systems. Infections with aerobic and anaerobic micro-organisms can cause edema, erythema, lymphadenopathy, lymphangitis, sepsis, necrosis and even osteomyelitis. Bite wounds are normally characterized by a mix of animal oral cavity micro-organisms. Dog bites are grossly contaminated, and common organisms involved Staphylococcus, Streptococcus, Pasteurella, Moraxella, and Corynebacterium.

Treatment includes appropriate prophylactic antibiotics and repeats cautiously surgical debridements. Closure of dog bites has largely been considered contraindicated due to the increased possibility of infection; however, this management remains controversial. Wounds at risk for infection should not undergo primary closure. Wound cleaning and debridement are key to reducing infection risk. Some patients may need rabies post-exposure prophylaxis. A dose of human rabies immune globulin (HRIG) and rabies vaccine will be given on the day of the rabies exposure, and then a dose of vaccine given again on days 3, 7, and 14.

Schrottner et al., in 2013, first reported a horse bite wound in a 2-year-old girl treated successfully with the semi-occlusive technique [12]. Despite of repeated isolation of bacteria that might cause wound infections, there were no signs of advancing infection or inflammation. In the semi-occlusive dressing, proceeding infections are not reported despite bacterial colonization. [13]. We report the second case of a fingertip amputation after an animal bite, which was treated successfully by a semi-occlusive dressing technique.

The phenomenon of self-regeneration of injured extremities is common in nature. The formation of a blastema, an aggregation of multipotent progenitor cells for the new digit or limb, is common in amphibian regeneration. However, little is known about the regeneration ability in humans, confined to the fingertips and hamstring tendons. Hamstring tendons regenerated in the majority of patients after ACL reconstruction. The majority of the hamstring tendon regeneration was found to occur between one month and one year after harvest [14]. 

This regenerative phenomenon is possible in a special micro-environment furnished by the wet chamber that the occlusive dressing helps create around the open wound. This occlusive environment is conducive to increased cell growth and re-epithelialization. The inflammation is very low, perhaps because the wet chamber allows for normal cellular hydration.

In the semi-occlusive dressing method, the dressing functions like a temporary "skin," making the finger painless [6]. It also provides a wet micro-environment that allows for skin regeneration, pulp, and even sensory ability. The wet environment facilitates and proliferates wound healing [15]. This regeneration begins immediately after the injury with a seal or a clot from tissue exudate and blood. Beneath this clot, epidermal cells proliferate and cover the trauma [15,16]. The fingertips regenerate fully, and the skin develops without scar formation. The micro-environment ultimately defines whether the formation of a blastema leads to the regeneration of the digit tip or healing with scar tissue as a failed regeneration attempt [16].

The regenerative tissue is able, under these circumstances, to provide ingredients that prevent inflammation or infection. Although the wounds were colonized with a wide range of bacteria and many positive probes for micro-organisms that may cause wound infections have been found [15], in the semi-occlusive treated wounds, the infection rates are lower (2%) than the infection rates with conventional gauze dressings (7%) )[17]. There is no report of a clinically relevant infection in several large series of semi-occlusive treated fingertip injuries [13,15]. The absence of infection is significant in contaminated wounds such as animal bites [6,10,13]

Compared to surgical interventions usually done for such injuries, there is no need for a hospital stay, immobilization, surgical complications, and donor site morbidity in the semi-occlusive dressing technique. However, one drawback may be the longer healing time [18]. Compared to conservative treatment, Hoigne et al. are convinced that the semi-occlusive treatment is different from secondary wound healing, as it is regenerative healing [10]. Almost scar-free healing occurs during semi-occlusive treatment, with the formation of an average of about 90% of the original soft tissue thickness and restoration of good sensibility in the injured area. In addition to the subcutaneous tissue formation, papillary lines develop as a sign of a dif­ferentiated regeneration of the skin [10].

This conservative method of semi-occlusive dressing has been used with successful outcomes in fingertip Allen I, II, and III amputations [1,6,10,13,19], and there are reports in the literature with satisfactory results even in Allen IV fingertip injuries [8].

## Conclusions

Semi-occlusive dressing technique is an effective management strategy in Allen type III fingertip amputations, especially an animal bite where primary closure is not recommended. Regeneration of the fingertip with satisfactory aesthetic and functional results regarding the soft-tissue thickness and pulp, nail complex and sensation can be achieved without signs of infection. 
